# Establishing Human Lacrimal Gland Cultures with Secretory Function

**DOI:** 10.1371/journal.pone.0029458

**Published:** 2012-01-13

**Authors:** Shubha Tiwari, Mohammad Javed Ali, Murali M. S. Balla, Milind N. Naik, Santosh G. Honavar, Vijay Anand P. Reddy, Geeta K. Vemuganti

**Affiliations:** 1 Sudhakar and Sreekant Ravi Stem Cell Biology Laboratory, Professor Brien Holden Eye Research Centre, L. V. Prasad Eye Institute, Hyderabad, India; 2 Ophthalmic Plastic Surgery, Orbit and Ocular Oncology, L. V. Prasad Eye Institute, Hyderabad, India; 3 Ophthalmic Pathology Services, Professor Brien Holden Eye Research Centre, L. V. Prasad Eye Institute, Hyderabad, India; 4 School of Medical Sciences, University of Hyderabad, Hyderabad, India; University of Southern California, United States of America

## Abstract

**Purpose:**

Dry eye syndrome is a multifactorial chronic disabling disease mainly caused by the functional disruptions in the lacrimal gland. The treatment involves palliation like ocular surface lubrication and rehydration. Cell therapy involving replacement of the gland is a promising alternative for providing long-term relief to patients. This study aimed to establish functionally competent lacrimal gland cultures *in–vitro* and explore the presence of stem cells in the native gland and the established *in-vitro* cultures.

**Methods:**

Fresh human lacrimal gland from patients undergoing exenteration was harvested for cultures after IRB approval. The freshly isolated cells were evaluated by flow cytometry for expression of stem cell markers ABCG2, high ALDH1 levels and c-kit. Cultures were established on Matrigel, collagen and HAM and the cultured cells evaluated for the presence of stem cell markers and differentiating markers of epithelial (E-cadherin, EpCAM), mesenchymal (Vimentin, CD90) and myofibroblastic (α-SMA, S-100) origin by flow cytometry and immunocytochemistry. The conditioned media was tested for secretory proteins (scIgA, lactoferrin, lysozyme) post carbachol (100 µM) stimulation by ELISA.

**Results:**

Native human lacrimal gland expressed ABCG2 (mean±SEM: 3.1±0.61%), high ALDH1 (3.8±1.26%) and c-kit (6.7±2.0%). Lacrimal gland cultures formed a monolayer, in order of preference on Matrigel, collagen and HAM within 15–20 days, containing a heterogeneous population of stem-like and differentiated cells. The epithelial cells formed ‘spherules’ with duct like connections, suggestive of ductal origin. The levels of scIgA (47.43 to 61.56 ng/ml), lysozyme (24.36 to 144.74 ng/ml) and lactoferrin (32.45 to 40.31 ng/ml) in the conditioned media were significantly higher than the negative controls (p<0.05 for all comparisons).

**Conclusion:**

The study reports the novel finding of establishing functionally competent human lacrimal gland cultures *in-vitro*. It also provides preliminary data on the presence of stem cells and duct-like cells in the fresh and *in-vitro* cultured human lacrimal gland. These significant findings could pave way for cell therapy in future.

## Introduction

The stability and integrity of the ocular surface depends greatly on the stability of the tear film that covers the anterior surface of the eye. The tear film has three basic layers - the outer thin lipid layer secreted by the meibomian glands, the middle bulk of aqueous layer secreted by the lacrimal gland and the inner mucinous layer secreted by the conjunctival goblet cells. Collectively, these three layers of the tear film perform a number of important physiological functions [1]: it keeps the cornea wet allowing gaseous exchange between the environment and the epithelium, it provides a clear and regular optical surface for sharp image focusing on the retina, it clears the debris from the ocular surface and protects it from microbial invasion. Deficiency and loss of tear film integrity, atrophy of the lacrimal gland or apoptosis of the secretory epithelial cells due to hormonal imbalance, environmental changes, autoimmune pathologies or radiotherapy induces pathological changes in the gland and leads to a chronic disabling condition called the dry eye syndrome (DES).

The 2007 International Dry Eye Workshop (DEWS) report estimated the global prevalence of DES to be between 5% to over 35% at various ages (21 yr to >65 yr) [2]. Clinically, chronic dry eye causes a significant drop in contrast sensitivity and visual acuity leading to degraded performance in routine vision related activities like driving, reading [3], [4]. The signs and symptoms include ocular dryness, grittiness, burning and foreign body sensation, redness and blurred vision that clears on blinking [5]. Over time the loss of tear film integrity induces corneal epithelial irregularities and epithelial defects [6] with higher risks of secondary infection [7]. The pathological features of dry eye include lymphocytic infiltration of the lacrimal gland [8], reversible squamous metaplasia [8], apoptosis of secretory epithelial cells, loss of α-smooth muscle actin and tenascin C expression in the myoepithelial cells indicating loss of function [9]. Together these contribute to reduced tear secretion and result in the signs and symptoms of dry eye. Biochemically, there is hyperosmolarity of the tear film either due to reduced tear production or excessive tear evaporation from the ocular surface causing a reduction in tear film thickness from (mean ± SD) 6.0±2.4 µm in normal subjects to about 2.0±1.5 µm in dry eye patients [10].

Current treatment for dry eye primarily involves the use of lubricating eye drops or pharmacological stimulation of tears secretion [7], [11]. However, these treatment modalities provide only temporary relief and have the inherent drawbacks of associated side effects and suboptimal results due to loss of secretory function of the gland [7]. In severe cases, especially in those with permanent damage to lacrimal gland, there arises a need to replace the gland and restore its functionality using appropriate cell therapy. To achieve this long-term goal it is important to establish and evaluate functionally competent *in-vitro* cell culture system.

Animal studies [12], [13], [14] have successfully demonstrated the establishment of *in-vitro* lacrimal gland cell cultures, using different media and scaffolds [12], [15]. However, work on human *in-vitro* lacrimal gland culture is scarce [16]; possibly due to paucity of fresh tissues and the fragile nature of these cultures. Recent studies have shown the presence of stem cells in exocrine glands like salivary [17], pancreas [18], [19], prostate [20] and breast [21], [22]. These reports have prompted investigations on the potentials of using *in-vitro* cultured cells for regenerative therapy with promising outcomes. However in the case of lacrimal gland, there is only preliminary report on the presence of stem cells in the mouse lacrimal gland [23], [24] and, to the best of our knowledge, none investigating the presence of stem cells in human lacrimal gland. With this background in mind, the present study had two important aims: 1) to establish functionally competent human lacrimal gland cultures *in-vitro* and 2) investigate the presence of stem cells in the native tissue as well as in the established *in-vitro* cultures.

## Materials and Methods

### Chemicals

HepatoSTIM culture media (BD biosciences, San Jose, CA, USA), Dulbecco's Modified Eagle's Medium (DMEM) - Ham's F12 (Sigma Aldrich, St Louis, MO,USA) Fetal calf serum (FCS) (Hyclone), bovine serum albumin (BSA) (Sigma Aldrich, St Louis, Mo, USA) penicillin, streptomycin, gentamysin and amphotericin B, epidermal growth factor (Sigma Aldrich, St Louis, MO, USA), L-glutamine (2 mM) (Sigma Aldrich, St Louis, MO, USA), Matrigel® basement matrix (BD Biosciences, San Jose, CA, USA), collagen I gel (Sigma Aldrich, St Louis, MO, USA), collagnease (Invitrogen, Carlsbad, CA, USA), hyaluronidase (Invitrogen, Carlsbad, CA,USA), Hank's Balanced Salt Solution (HBBS), TRIzol (Invitrogen, Carlsbad, CA,USA), Superscript first strand synthesis system for RT-PCR (Invitrogen, Carlsbad, CA, USA) anti- E-cadherin antibody (Chemicon, Temecula, CA, USA), anti-ABCG2 (BD Biosciences, San Jose, CA, USA), anti c-kit (Millipore, Temecula, CA; Dako, Glostrup, Denmark), anti-p63 (Dako, Glostrup, Denmark) anti-CD133 (Miltenyi Biotech), anti-EpCAM (BD Biosciences, San Jose, CA, USA) anti- lysozyme (Abcam), anti- lactoferrin (Millipore, Temecula, CA, USA), anti-scIgA (Dako, Glostrup, Denmark), anti- GFAP (Dako, Glostrup, Denmark), anti-S100 protein (Dako, Glostrup, Denmark) anti CD90 (eBioscience), anti-vimentin (Dako, Glostrup, Denmark), anti-CK3/12 (Dako, Glostrup, Denmark), Aldefluor Assay Kit (Stem Cell Technology, Durham, NC,USA), Fluroscein Isothiocynate (Invitrogen, Carlsbad, CA,USA), Alexa Fluor 488 (Invitrogen, Carlsbad, CA, USA), Phycoerytherin (eBioscience), human lactoferrin ELISA kit (AssayPro, MO, USA), human lysozyme ELISA kit (AssayPro, MO, USA), human IgA ELISA kit (Immunology Consultants Laboratory, Inc, Newberg, OR, USA).

### Human Tissue Source

The study was conducted at the L V Prasad Eye Institute (LVPEI), Hyderabad. The use of human tissue was approved by the Institutional Review Board (IRB) and is in accordance with the tenets of the Declaration of Helsinki. Fresh human lacrimal gland was harvested, after obtaining written informed consent, from patients undergoing exenteration surgery. The lacrimal gland was immunohistochemically evaluated to be free from any underlying pathology. The fresh gland was collected in FCS rich DMEM-Ham's F-12 media supplemented with antibiotics and transported to the lab where it was immediately taken for processing.

### Preparation of human amniotic membrane

The use of human amniotic membrane has been approved by the IRB of LVPEI, Hyderabad. Human amniotic membrane (HAM) was prepared according to the protocol published by Kim *et.al* [25] Following written informed consent, human placenta was obtained at the time of normal cesarean delivery at the Fernandez Hospital, Hyderabad. The placenta was transported to the Ramayamma International Eye Bank (LVPEI, Hyderabad) and processed under sterile conditions using saline/ringer lactate solution. The amnion was separated from the chorion by peeling and attached onto sterile nitrocellulose paper and cut into 2.5×5.0 cm pieces. The amniotic membrane pieces were stored in glass vials containing DMEM and stored at −70°C. Just prior to use, it was thawed at 37°C for 30 minutes. HAM was de-epithelized by incubating with 0.25% trypsin–EDTA for 30 minutes at 37°C followed by thorough washing with PBS to remove the epithelial layer [26].

#### Establishment of primary culture

Fresh lacrimal gland was washed with HBBS to remove red blood cells. The gland was chopped into small bits using a scalpel blade (#21). The tissue mince was then incubated with the enzyme cocktail of collagenase (130 units per ml) and hyaluronidase (300 units per ml) for 90 minutes at 37°C with intermittent shaking. At the end of the incubation period, the suspension was filtered through a 75 µm cell sieve and the cell pellet obtained by centrifugation at 1500 rpm for 20 min. The cells were seeded on uncoated tissue culture dishes in DMEM-Ham's F12 culture media supplemented with 10% FCS and antibiotics.

The heterogeneous mix of isolated cells was separated based on their preferential adhesion to uncoated tissue culture dishes. The fibroblasts attached preferentially to the uncoated tissue culture dishes and the epithelial clumps, still in suspension at the end of around 2 hours, were aspirated and plated on Matrigel™, collagen I coated dishes and on denuded HAM. The media used for the culture of fibroblasts was DMEM- Ham's F-12 supplemented with 2 mM L-glutamine, antibiotics and 10% FCS. For the culture of lacrimal acinar cells, HepatoSTIM™ culture media was used. HepatoSTIM® is a commercially available fully defined, serum free media based on the formulation of Williams E Media [27] and contains supplements like dexamethasone, insulin-transferrin-selinium (ITS) and EGF. The media was further supplemented with 2 mM L-glutamine, penicillin, streptomycin, 5 ng/ml EGF and 10% FCS for the first three days. At the end of the three days/at first media change, FCS was completely eliminated from the media and the concentration of EGF increased to 50 ng/ml.

#### Characterization of native lacrimal gland and in-vitro lacrimal gland culture

The native lacrimal gland and 14–18^th^ day *in-vitro* cultures of human lacrimal gland, growing as a monolayer on Matrigel™ were characterized by immunohistochemistry, immunocytochemistry, flow cytometry, reverse-transcriptase polymerase chain reaction (RT-PCR) and enzyme linked Immunosorbent assay (ELISA).

### Immunohistochemistry

Expression of markers like Pan-cytokeratin (AE1/AE3), vimentin, p63, ∝-SMA, GFAP, S-100, lysozyme and c-kit protein was evaluated in formalin fixed, paraffin embedded sections of fresh lacrimal gland. The sections were also stained with hematoxylin & eosin (H&E) as well as periodic acid schiff's (PAS) to visualize the histology of the gland.

The fresh gland was fixed with 10% fresh formalin and embedded in paraffin. Thin 3 µm sections were taken on silane coated glass slides and used for immunostaining. Briefly, the paraffin embedded sections were deparaffinized at around 70°C and then in xylene series. The sections were rehydrated in alcohol series and then in distilled water followed by 1X PBS. The endogenous peroxidase activity was blocked using methanol and hydrogen peroxide and the antigen retrieval done using Tris-EDTA buffer (pH 9). After appropriate washings with PBS and blocking with 2.5% BSA, the sections were incubated with the primary antibody in a moist chamber for 2 hours at room temperature followed by secondary antibody (polymer HRP) incubation for 30 minutes at room temperature. DAB substrate was added to the section to allow color development for 10 minutes. This was followed by counterstaining with hematoxylene and then mounting in DPX. The sections were visualized under a light microscope.

The source of the antibodies used and the appropriate dilutions are summarized in Table 1.

**Table 1:** List of antibodies and dilutions used for immunohistochemistry.

S. No.	Antibody	Dilution	Company
1.	p63	Neat	Dako
2.	∝-SMA	Neat	Dako
3.	GFAP	Neat	Dako
4.	S-100 protein	Neat	Dako
5.	Lysozyme	1∶100	Abcam
6.	c-kit	1∶100	Dako
7.	Pan-cytokeratin (AE1/AE3)	1∶50	Dako
8.	Vimentin	1∶200	Dako

### Immunocytochemistry

The *in-vitro* cultures of lacrimal gland at day 14–18 were immunostained for epithelial markers like cytokeratin 3/12, E-cadherin, p63; myoepithelial markers like S100, GFAP; and mesenchymal markers vimentin and CD 90. In addition, the cells were also immunostained with ABCG2 and secretory protein lysozyme. Briefly, the cells were fixed with 4% fresh paraformaldehyde (PFA) for 10 minutes, followed by permeabilization with 50% methanol for 20 minutes for intracellular markers. The cells were then incubated with appropriate dilutions of the primary antibody for 2 h at room temperature.

Secondary antibodies like Alexa Fluor 488, FITC and PE were used against the respective immunoglobulins of the primary antibody at 1∶200 dilution. The incubation time was 45 min. Nuclear staining was done with 4,6-diamidino-2-phenylindole (DAPI) or propidium iodide (PI). The coverslips were mounted in 50% glycerol and the images acquired using Carl Zeiss Laser Scanning Microscope LSM 510.

The source of the antibodies used and the appropriate dilutions are summarized in Table 2, 3 and 4.

**Table 2:** List of antibodies and dilutions used for immunocytochemistry.

S. No.	Antibody	Dilution	Company
1	E- cadherin	1∶100	Millipore
2.	Cytokeratin 3/12	1∶100	Millipore
3.	CD90	1∶200	eBiosciences
4.	Vimentin	1∶100	Dako
5.	GFAP	Neat	Dako
6.	S-100 protein	Neat	Dako
7.	Lysozyme	1∶100	Abcam

**Table 3:** List of antibodies and dilutions used for flow cytometry.

S. No.	Antibody	Dilution	Company
1	ABCG2	1∶100	BD Biosciences
2.	c-kit	1∶100	Millipore
3.	E-cadherin	1∶100	Millipore
4.	CD90	1∶200	eBiosciences
5.	EpCAM	1∶100	BD Biosciences
6.	CD133	1∶20	Miltenyi Biotech

**Table 4:** List of secondary antibodies and dilutions.

S. No.	Antibody	Dilution	Company
1.	Alexa Fluor 488	1∶200	Invitrogen
2.	PE	1∶200	eBiosciences
3.	FITC	1∶200	Invitrogen

### Flow Cytometry

Lacrimal gland cells, freshly isolated from the gland as well as 14–18 days and 21–25 days post *in-vitro* culture, were evaluated by flow cytometry to detect the number of cells positive for epithelial markers like E-cadherin, EpCAM and mesenchymal markers like CD90. Putative stem cell markers like ABCG2, c-kit were also used to investigate the presence of stem cell compartment. Expression of CD133, which has recently been recognized as a stem cell marker/marker for glandular epithelium [28], was also evaluated.

Cells were isolated from the human lacrimal gland by enzymatic digestion as described previously in the study. Cells growing as monolayers on Matrigel™ were trypsinized using 0.25% trypsin-EDTA (TE) and used for evaluation of marker expression by flow cytometry.

Briefly, 1×10^6^ cells were fixed with 4% fresh PFA for 10 minutes, blocked with 5% BSA and incubated with appropriate dilutions of primary antibody for 2 hours at room temperature. The cell pellet was washed with PBS and then incubated with 1∶200 dilutions of appropriate secondary antibodies for 45 minutes. At the end of this time period, the pellet was washed with PBS, resuspended in 500 µl of FACS buffer and acquired on BD FACS ARIA™ Special Order System. Appropriate controls were used for the experiment. A total of 20000 to 50000 events were acquired for analysis. The analysis was done using BD FACS DiVa™ software.

The source of the antibodies used and the appropriate dilutions are summarized in Table 2, 3 and 4.

### Aldefluor Assay

ALDEFLUOR® fluorescent reagent system (Stem Cell Technologies) provides a novel method for identification of stem and progenitor cells based on their expression of enzyme aldehyde dehydrogenase1 (ALDH). The fluorescent ALDEFLUOR® reagent diffuses freely into the cells and acts as a non-toxic substrate for ALDH. The fluorescent reaction product that accumulates in the cells can be measured in the green channel of a standard flow cytometer and it correlates directly to the ALDH activity in the cell. With this assay, stem and progenitor cells are identified as cells with higher expression of ALDH1.

For the assay, 1×10^6^ cells/ml (freshly isolated from the lacrimal gland as well as post trypsinization from the cultured monolayer on day 14–18 and day 21–25) were taken and divided into two groups: control and test. To the control tube 5 µl of ALDH inhibitor DEAB was added. 5 µl/ml of activated ALDEFLOUR® substrate was added to the test group and immediately half of the cell suspension was transferred to the control tube. Both the test as well as the control sample was incubated at 37°C for 30 to 60 min. At the end of the incubation period the supernatant was removed after centrifugation and the cells resuspended in 0.5 ml of ALDEFLUOR® Assay Buffer and the fluorescence measured in the green channel of FACS ARIA™ Special Order System.

### Reverse- Transcriptase Polymerase Chain Reaction

Total RNA was extracted from the freshly isolated as well as cultured cells using the TRIzol® reagent according to the manufacturer's instructions. The quality of the RNA isolated was checked by visualization on agarose gel. 2 µg of RNA was used for cDNA synthesis per 25 µl of the reaction volume using the Superscript™ First Strand Synthesis System for RT-PCR according to the manufacturer's instructions. The primer sequences used for reverse transcriptase polymerase chain reaction are summarized in Table 5.

**Table 5:** Primer sequences used for RT-PCR.

S. No.	Name	Sequence	Product Size (bp)
1.	Lactoferrin - Forward	CAGACCGCAGACATGAAACT	479
	Lactoferrin- Reverse	TTCAAGAATGGACGAAGTGT	
2.	Lysozyme - Forward	CTCTCATTGTTCTGGGGC	350
	Lysozyme - Reverse	ACGGACAACCCTCTTTGC5	
3.	scIgA - Forward	AATGCTGACCTCCAAGTGCTAAAG	242
	scIgA - Reverse	ATCACCACACTGAATGAGCCATCC	
4.	GAPDH - Forward	CAGAACATCATCCCTGCATCCACT	250
	GAPDH - Reverse	GTTGCTGTTGAAGTCACAGGAGAC	

PCR amplification was carried out using the Applied Biosystems Veriti 96 well thermal cycler. The reaction was stopped at 35 PCR cycles. The amplified products were visualized on agarose gel and the product size estimated.

### Measurement of secretory components

Free secretory products of lacrimal acinar cells like scIgA, lactoferrin and lysozyme were detected in the culture supernatant of day 6–7, day 14 and day 21 cultures according to the manufacturer's instructions. All the reagents used were supplied as a part of the ELISA kit. A standard curve was generated for each experiment performed.

Briefly, 50 µl of the standard protein or culture supernatant was added to the wells of polypropylene U bottom ELISA plates and incubated for 2 hours at room temperature. The plates were washed at least five times with the wash buffer (supplied with the kit) ensuring complete removal of the liquid at each step. 50 µl of biotinylated primary antibody (lysozyme/lactoferrin/scIgA) (1∶100 dilution) was added to each of the sample-coated wells and incubated for one hour at room temperature followed by thorough washing with the wash buffer. 50 µl of streptavidine-peroxidase conjugate was added to each well and incubated for 30 minutes. At the end of the incubation period, the wells were washed thoroughly and incubated with 50 µl/well of the chromogen substrate for 10 minutes at room temperature. The reaction was stopped by adding 50 µl of stop solution to each well and a color change from blue to yellow is noted. The optical density (OD) was then measured immediately at 450 nm on ELISA microplate reader (BioRad iMark™ Microplate Reader).

### Statistical Analysis

Values are expressed as mean of triplicate readings ± SEM unless otherwise indicated. The statistical test used was two-way ANOVA with post hoc Tukey test. Statistical package SPSS Version 19 was used for analysis and graphs plotted using Microsoft excel. The results were statistically compared with fresh media (as a negative control) and also with each group and were considered as statistically significant if p≤0.05.

## Results

After IRB approval and informed consent, 22 samples of fresh lacrimal gland tissue were harvested from exenterated specimens for orbital malignancies. After histological confirmation of normal lacrimal gland and exclusion of any underlying pathology, 18 were included for the study. Cultures were established in 14 of the 18 samples while 4 samples were used for FACS analysis after enzymatic digestion.

### Establishment of human lacrimal gland primary culture

**Figure 1:**
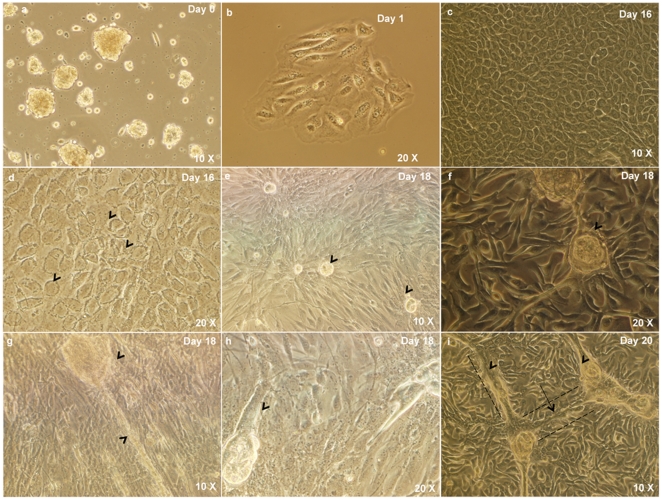
Establishment of human lacrimal gland primary cultures. a) Heterogenous cell population isolated after enzymatic digestion of the lacrimal gland. b) Cell clumps adhere to the substrate as discrete islands and show initiation of proliferation within 1–3 days. c) The islands proliferate and form a confluent monolayer within 15–20 days. d) The cells in the monolayer show thin cytoplasmic border, vesicular nucleus and granularity in the cytoplasm. e–f) Spherules are formed by day 16–18 (arrow head). g–i) Cord-like connections (arrow head) are seen to develop between the spherules.

The enzymatic digestion of the freshly harvested lacrimal gland tissue yielded a heterogeneous population of cells comprising of clumps of epithelial cells, fibroblasts, single cells of larger size and some red blood cells (Figure 1a).

On initially plating the isolated cells on uncoated tissue culture dishes, the fibroblasts settled down and adhered within 2 hours at the end of this time period, the floating cells were aspirated and seeded on dishes coated with Matrigel™, collagen I or on denuded HAM.

The epithelial cells from the suspension adhered to Matrigel™, collagen I coated dishes and denuded HAM with initiation of cell proliferation within 1–3 days (Figure 1b). Preferential adhesion of the cell clumps, optimal proliferation and morphology of the epithelial cells was seen on Matrigel™. Within 15–20 days, the epithelial islands expanded to form a monolayer in all the three substrates (Figure 1c). The cells showed thin cytoplasmic borders, polygonal shape, vesicular nucleus and granular cytoplasm (Figure 1d). The cultures on Matrigel showed sustained epithelial morphology for 30–35 days. (Panel showing day 14 epithelial cell growth on uncoated dishes, denuded HAM, collagen 1 and Matrigel™ coated dishes is included as Figure S1).

In addition, there was formation of ‘spherules’ (Figure 1e, f) in all matrices by day 16–18. The cultures also showed development of cord-like cellular structures between two spherules suggesting an attempt towards formation of duct-like structures (Figure 1g–i).

**Figure 2:**
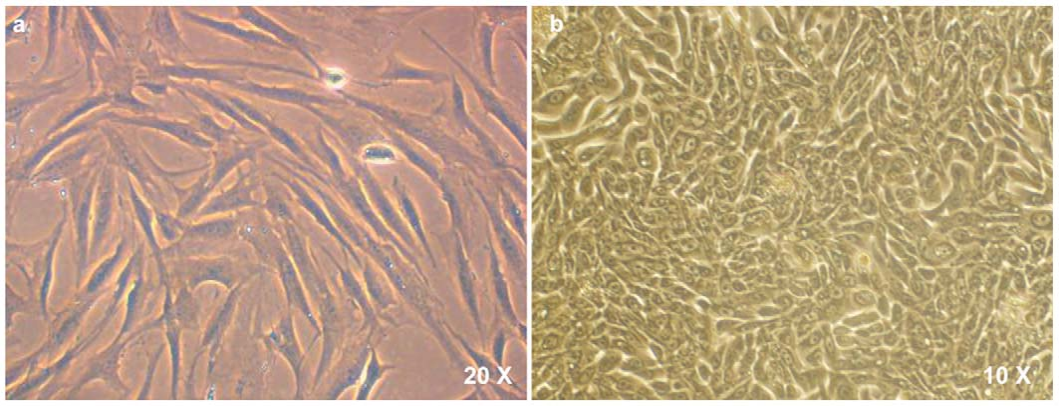
Other cell types in culture. a) Spindle shaped cells with slight granularity in their cytoplasm and distinct nucleus, are seen on uncoated culture dishes and these attain confluence within 5–7 days. b) Oval and plump cells that organize themselves in whorls are also seen. These may be myoepithelial in nature.

On uncoated tissue culture dishes, there was predominance of spindle cells that form a confluent monolayer by around 5–7 days. The cells were spindle shaped, with slight granularity in their cytoplasm and distinct nucleus (Figure 2a). In addition, the culture also showed the presence of a third type of cellular morphology: oval and plump cells that organized themselves in whorls (Figure 2b). These could be myoepithelial in nature.

A flow cytometric comparison was also made of the heterogeneous cell population growing on uncoated, collagen 1 and Matrigel™ coated dishes. The day 14 data shows that about 85.1±4.9% of the cells growing on uncoated tissue culture dishes are positive for mesenchymal marker CD90 while only 0.65±0.35% were positive for epithelial marker EpCAM; on collagen 1 coated dishes, 16.7±0.85% of the cells showed CD 90 positivity while 1.2±0.3% were EpCAM positive; as for Matrigel, 13.3±10.2% of the cells were mesenchymal (CD90 positive) while 2.2±1.7% were epithelial (EpCAM positive) (Table S1).

### Immunophenotyping of native and cultured lacrimal gland cells

#### Immunohistochemistry

**Figure 3:**
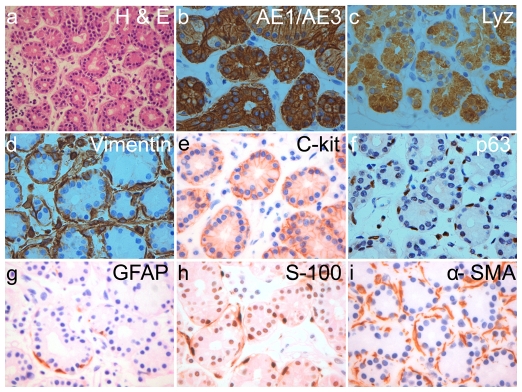
Immunohistochemistry on normal human lacrimal gland. H&E staining shows the normal histology of the lacrimal gland. Marker staining pattern shows localization of pan-cytokeratin (AE1/AE3) and lysozyme (Lzy) in the cytoplasm of the acinar cells while c-kit is seen in the plasma membrane of acinar cells. p63, glial fibrillary acidic protein (GFAP), S-100 protein and ∝-SMA localize in the myoepithelial cells enveloping the acinar cells. Vimentin is seen in the myoepithelial cells and also in some of the acinar cells. All images are at 40× magnification except H&E which is at 10×.

The formalin fixed, paraffin embedded human lacrimal gland tissues, on hematoxylene and eosin staining show the typical tissue architecture of human lacrimal gland (Figure 3a). These paraffin embedded sections showed immunoreactivity for pan-cytokeratin, lysozyme, vimentin, c-kit, p63, ∝-SMA, GFAP and S-100. The staining pattern reveals localization of pan-cytokeratin (Figure 3b) and secretory protein lysozyme (Figure 3c) mostly in the acinar cells with very few ductal cells showing faint positivity. Vimentin localized in the myoepithelial cells around the acinar cells and in the fibroblasts of the stroma. Few acinar cells also show vimentin positivity (Figure 3d). c-kit expression was seen as a membrane marker in the cell membrane of the acinar cells (Figure 3e) while p63, GFAP, S-100 and ∝-SMA (Figure 3f–i) was found in the cells enveloping the acinar cells (myoepithelial cells).

### Immunocytochemistry

**Figure 4:**
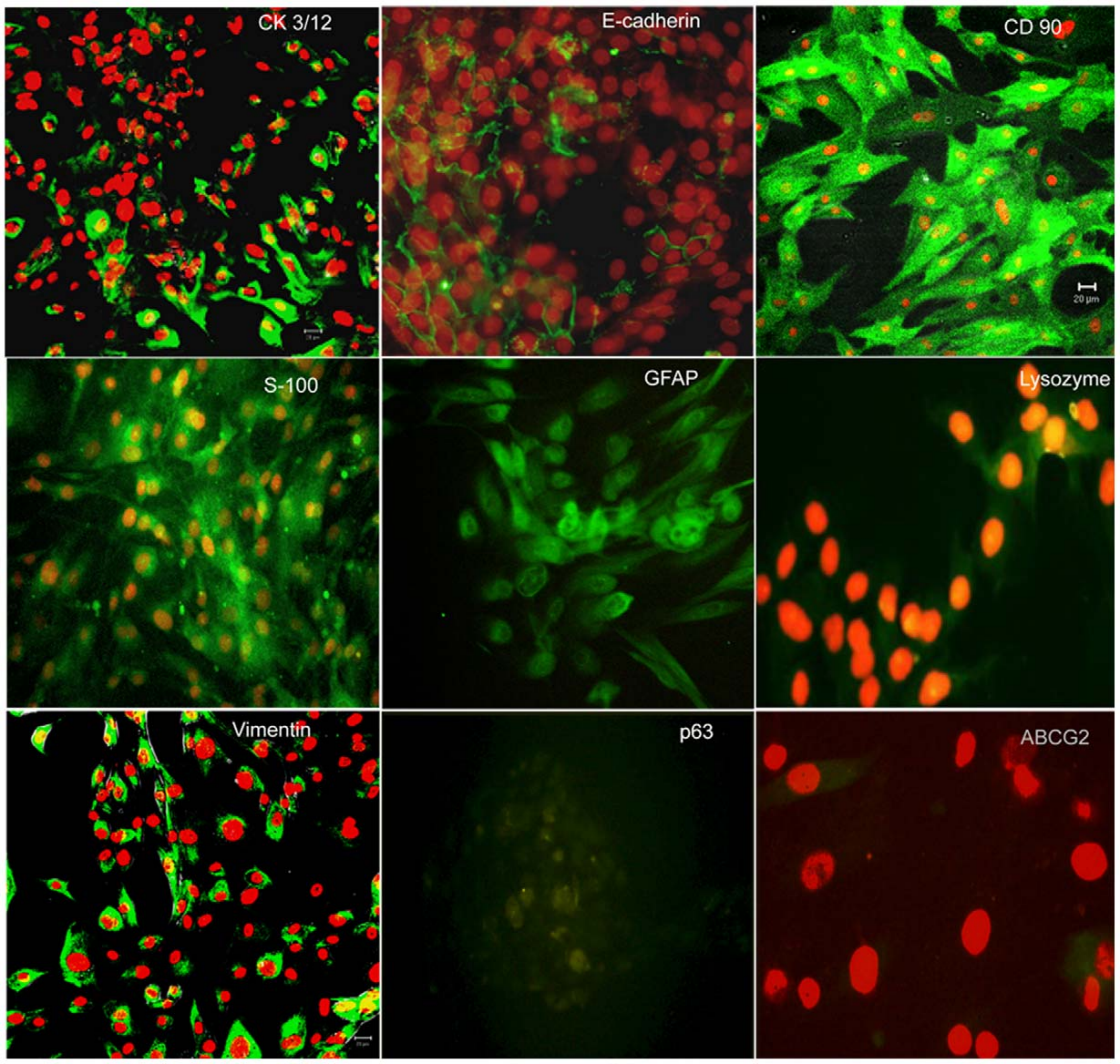
Immunocytochemistry on in-vitro cultured human lacrimal gland cells. Cells with epithelial morphology stain positively with E-cadherin, CK3/12, lysozyme and p63; oval and plump cells stain positive for myoepithelial markers GFAP and S100 protein while the spindle shaped cells are seen to be positive for mesenchymal markers CD90 and vimentin. Some cells also show immunopositivity for ABCG2. Secondary antibody uses is fluoresceine isothiocyanate (green) and the counter-stain is propidium iodide (red).

The cultures showed a heterogeneous population of cells with immunoreactivity for epithelial, myoepithelial as well as mesenchymal markers. The cells with epithelial morphology show positivity for CK3/12, p63, E-cadherin and lysozyme. CK3/12 and lysozyme localized in the cytoplasm of the cells, p63 in the nucleus while E-cadherin was seen to localize around the plasma membrane and between the epithelial cells. Some of the cells also showed immunopositivity for ABCG2 (Figure 4).

The spindle shaped cells were positive for mesenchymal markers CD90 and vimentin. The oval and plump cells were immunoreactive for GFAP and S-100 protein, which may be indicative of their myoepithelial/ductal origin (Figure 4).

### Flow Cytometry

**Figure 5:**
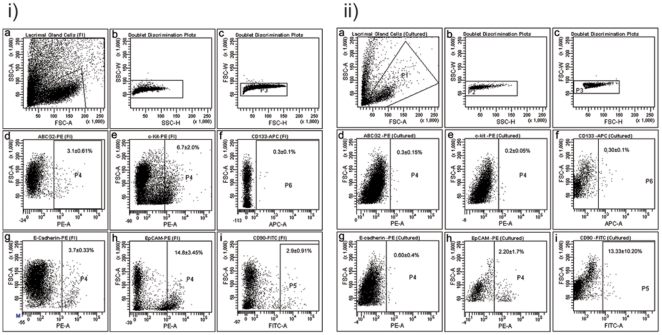
Flow Cytometry Data. i) Flow cytometric profile of freshly isolated (FI) cells. ii) Flow cytometric profile of cells 14–18 days post *in-vitro* culture.

The FACS analysis of freshly isolated cells from the human gland showed that 14.8±3.45% of the cells were positive for epithelial marker EpCAM, 2.9±0.91% for mesenchymal marker CD90 and 3.7±0.33% positive for E-cadherin indicative of epithelial/epithelial progenitor nature. Cells in this heterogeneous mix also showed immunoreactivity towards stem cell markers like ABCG2 (3.1±0.61%) and c-kit (6.7±2.0%). CD133 positivity was seen in 0.3±0.1% of the cells (Table 6) (Figure 5i).

**Table 6:** Flow cytometry data: Percentage of various marker in freshly isolated, day 14–18 and day 21–25 of in-vitro culture of human lacrimal gland.

S. No.	Marker	% Expressed at t = 0 (Mean± SEM)	% Expressed at day 14 of *in-vitro* culture (Mean± SEM)	% Expressed at day 21 of *in-vitro* culture (Mean± SEM)
1.	ABCG2	3.1±0.61	0.3±0.15	0.2±0.13
2.	c-kit	6.7±2.0	0.2±0.05	0.13±0.03
3.	E-cadherin	3.7±0.33	0.6±0.4	0.45±0.25
4.	EpCAM	14.8±3.45	2.2±1.70	0.3±0.1
5.	CD90	2.9±0.91	13.3±10.20	30.25±3.35
6.	CD133	0.30±0.10	0.30±0.10	0.25±0.05

FACS analysis of cultured lacrimal gland cells also showed the presence of stem cells as well as differentiated cells in day 14–18 cultures. 2.2±1.7%of the cells were positive for EpCAM, 13.3±10.2% positive for CD 90. The cultures had a population of cells with positivity for stem cell markers accounting for 0.30±0.15% as ABCG2 positive and 0.20±0.05% as c-kit positive. CD133 expression was seen in 0.3±0.1% of the cells (Figure 5ii). By day 21–25 of *in-vitro* culture the number of cells expressing the stem cell markers ABCG2 and c-kit had reduced to 0.2±0.13% and 0.13±0.03% respectively while the expression of differentiated markers like CD90 had increased to 30.25±3.35%. This could possibly be and indication of the ongoing differentiation under *in-vitro* conditions (Table 6) (FACS plots not included for day 21–25).

### ALDEFLUOR Assay

**Figure 6:**
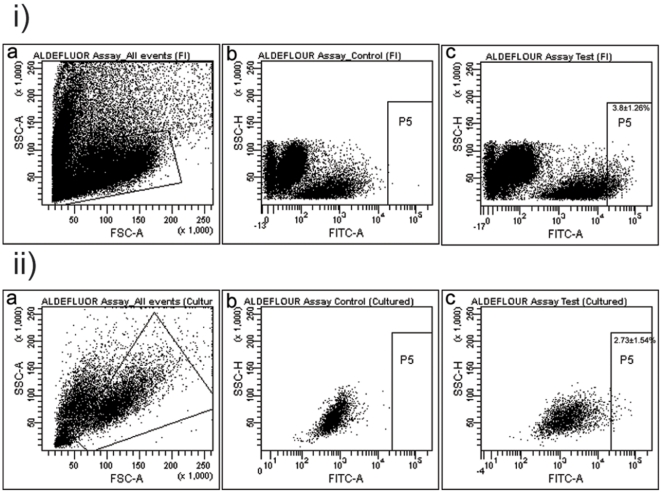
Aldefluor Assay. i) Flow cytometric data showing ALDH1 high cells in the freshly isolated (FI) cell population. ii) Flow cytometric data showing ALDH1 high cells in day 14–18 *in-vitro* cultures.

In the freshly isolated cells, 2.4% to 6.3% showed high ALDH1 activity (mean: 3.8±1.26%). Co-expression of high ALDH1 and ABCG2 was seen in 0.13±0.04%, and high ALDH1 and c- kit in 0.21%±0.02% of the cells (Table 7) (Figure 6i).

**Table 7:** ALDEFLUOR Assay.

S. No.	Marker	% Expressed at t = 0 (Mean± SEM)	% Expressed at day 14 of *in-vitro* culture (Mean± SEM)
1.	ALDH1 high	3.8±1.26	2.7±1.54
2.	ALDH high + c-kit	0.2±0.02	-
3.	ALDH high + ABCG2	0.1±0.04	-

The day 14–18 cultured lacrimal gland cells also showed a population with high ALDH activity accounting for 2.7±1.5% of the total population acquired (Figure 6ii) which decreases to 1.1±0.5% by day 21. Co-expression of high ALDH1 with ABCG2/c-kit positivity was not evaluated in the cultured cells since their level of expression in the native tissue itself was low to begin with. (FACS plots not included for day 21–25).

### C. Secretome Assessment

#### Reverse- Transcriptase Polymerase Chain Reaction

**Figure 7:**
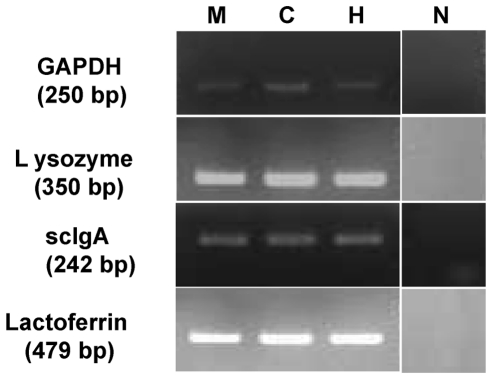
RT-PCR showing appropriate product bands for scIgA, lactoferrin and lysozyme in day 14 *in-vitro* cultures on all HAM (H), collagen I (C) and Matrigel™(M). Negative (N) panel shows no amplification product.

In order to confirm the presence of mRNA for the secretory proteins, lactoferrin, lysozyme and scIgA, in the cultured human lacrimal gland cells RT PCR was performed with specific primers. The cDNA synthesized by reverse transcription showed the expression of scIgA, lactoferrin and lysozyme (Figure 7) in the cells thereby confirming that the cultured cells retained their physiological ability to synthesize the secretory proteins.

### Enzyme Linked Immunosorbent Assay

**Figure 8:**
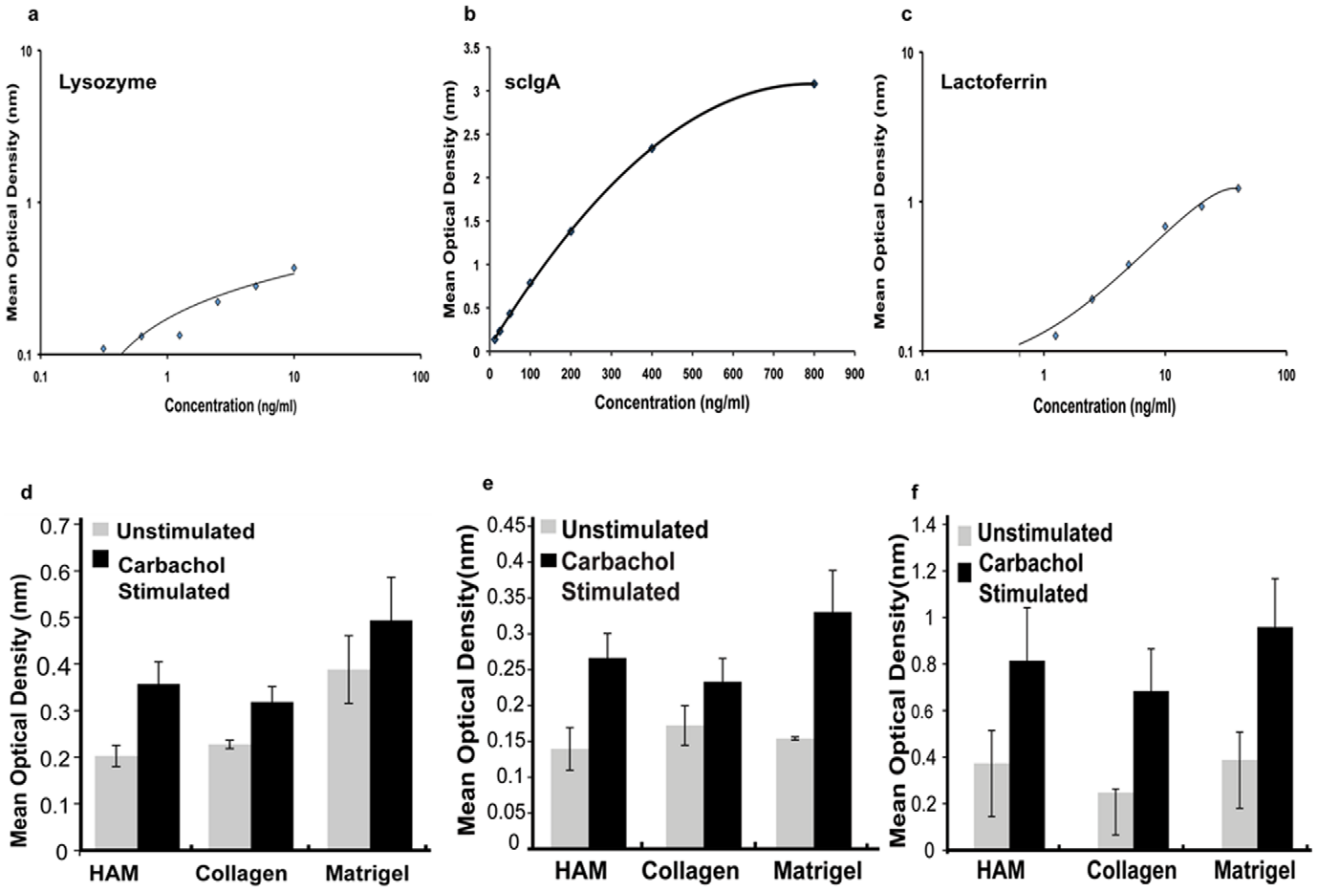
Secretome Assessment by ELISA. a–c): Standard calibration curve for lysozyme, scIgA and lactoferrin. d–f): Plot of mean optical density values for secreted proteins lysozyme, scIgA and lactoferrin on HAM, collagen and Matrigel™ pre and post carbachol stimulation.

The conditioned media of day 6–7 human lacrimal gland cultures showed the presence of scIgA, lactoferrin and lysozyme secreted by the acinar cells. The quantity of protein secreted into the conditioned media by the cells growing on each of the matrices was calculated using the standard calibration curves (Figure 8a–c).

The protein secretion was further augmented by treatment with 100 µM of carbachol for 30 min (Figure 8d–f). The main effect of carbachol stimulation was statistically significant for sc IgA (F(1, 8) = 15.07; p<0.01), lysozyme (F(1,8) = 5.86; p = 0.02) and lactoferrin (F(1,8) = 11.44; p<0.01) secretion.

The amount of protein secreted by the cells post carbachol stimulation on each of the three matrices is tabulated in Table 8.

**Table 8:** ELISA data: Secretion of tear proteins on various matrices post carbachol stimulation on day 6–7.

Tear Protein	HAM (ng/ml)	Collagen I (ng/ml)	Matrigel™ (ng/ml)
**Lysozyme**	5.78 to 33.94	0.21 to 18.34	24.36 to 144.74
**Sc IgA**	3.86 to 71.40	1.41 to 27.58	47.43 to 61.56
**Lactoferrin**	44.50 to 45.65	28.52 to 30.41	32.45 to 40.31

The cells cultured on Matrigel showed slightly higher levels of secretory proteins in the conditioned media (Figure 8d–f). However, the main effect of matrices and the interaction between matrices and carbachol stimulation was not statistically significant for scIgA (F (2,8) = 1.33; p = 0.27) and lactoferrin (F(2,8) = 0.86; p = 0.43) secretion; but showed significance for lysozyme secretion (F(2,8) = 5.0; p = 0.01).

The trend in protein secretion by the cultured cells at day 7, day 14 and day 21 was also evaluated. The results indicate that *in-vitro* tear protein secretion tends to increase from day 7 to day 14 and then declines by day 21. (Table S2).

This change over a time period from day 7 to day 21 was statistically analysed and found to be significant: scIgA (F(1,6) = 21.92; p<0.01); lysozyme (F(1,6) = 7.45; p<0.01) and lactoferrin (F(1,6) = 21.3; p<0.01).

## Discussion

Similar to the role of other exocrine glands of the body, the lacrimal gland plays a major role in lubricating the ocular surface epithelium. The susceptibility of the gland to immune mediated insults, radiation induced damage and age related atrophic changes, which cannot be satisfactorily managed with the current line of therapeutics, causes high incidences of morbidity in the population and raises a need for replacement therapy with functionally competent cells.

The two important steps towards cell therapy would include establishment of functionally competent lacrimal gland cultures with persevered secretory function and providing evidence for the existence of stem cells in the native tissue, which can be recruited to salvage the function of the damaged gland. The present study provides evidence for both. Our results show that the human lacrimal gland cells can be cultured *in-vitro* with retained secretory function (Figure 1, Figure 7 and Figure 8). In addition to the mere presence of differentiated cells (epithelial, myoepithelial and stromal), we also show the formation of ‘spherules’ with attempted duct-like connections (Figure 1 e–i) and the presence of cells with stem cell-like phenotype (ABCG2 & ALDH1 high cells) (Figure 5 and Figure 6) under our culture conditions.

While lacrimal gland cultures from rabbit [13], mice [14] and rat [12] sources are well established, similar evidence from humans is limited [16]. To the best of our knowledge, this study documents the first successful method of isolating and culturing functionally competent fresh human lacrimal gland cells using an enzyme cocktail of collagenase and hyaluronidase. The established cultures can be maintained *in-vitro* for 30–35 days. This method not only gives an adequate yield of differentiated cells, but also provides early evidence for the presence of putative stem cells in the tissues as suggested by the expression of stem cell makers ABCG2 (3.1±0.61%) and high ALDH1 (3.8%±1.26%).

The influence of culture condition on cell proliferation and maintenance of function, especially in the case of lacrimal gland cultures, has been well documented [29], [30], [31]. It is important that the right combination of substrate, media and growth factors be used to simulate an *in-vivo* mimicry of function. Animal lacrimal gland cultures have been established on collagen [30], Matrigel™ [12], [15], HAM [32] and on artificial matrices like polyethersulfone [33]. Our study with human tissues also suggest that a combination of Matrigel™/HAM/collagen as substrate and EGF supplemented HepatoSTIM™ media optimally supports the growth of different sub-populations of cells and helps them retain their characteristic marker expression (E-cadherin, CD90, S100, GFAP) as well as optimal secretory function (scIgA, lactoferrin and lysozyme secretion). However, morphological evidence and rate of cell proliferation seems to favor Matrigel™ as substrate even though this difference is not statistically significant. Another interesting feature noted in this study is morphologic evidence towards formation of spherules and ductioles. The formation of spheres has been reported in salivary gland cultures (salispheres) [34], prostate cultures (prostaspheres) [35] and a single study on spheroidal aggregation of rabbit lacrimal gland cells grown in microgravity environment of a rotary cell culture system [36]. The salispheres have been shown to have stem cells and these when transplanted into animal models of radiation-induced xerostomia could rescue the function of the gland [34]. Similarly, the prostaspheres on combined transplantation with embryonic day 17 urogenital sinus (which provides the mesenchymal niche) into nude mice could give rise to tissues with glandular prostate morphology [35]. We believe that the spheres formed under our culture conditions could be similar to the salispheres and prostaspheres in their cellular organization and further investigations in this are ongoing.

In order to fulfill the long-term goal of using the *in-vitro* expanded lacrimal cultures for rescue of function of damaged gland it is important that the secretory profile of these cells be evaluated. Yoshino *et.al.* [16] reported a study on human lacrimal gland using cadaveric tissue; however, the report does not document the complete secretory profile of the cultured cells. In contrast, the present study shows that *in-vitro* cultures of human lacrimal gland acinar cells can retain their functionality of secreting major tear proteins like lysozyme, scIgA and lactoferrin (Figure 7 and 8). This secretory profile has also been quantified by sandwich ELISA technique using the calibration curve generated for each of the three proteins. The calibration curve was generated in two sets of experiments in which readings were taken in triplicates and since the optical density values for the remaining three sets were very similar, the same calibration curve was used to estimate the protein quantity. Our results show that the secretion of these proteins ranges from 3.86 to 71.4 ng/ml of scIgA, 5.78 to 33.94 ng/ml of lysozyme and 44.5 to 45.65 ng/ml of lactoferrin on HAM, to 1.41 to 27.58 ng/ml of scIgA, 0.21 to 18.34 ng/ml of lysozyme and 28.52 to 30.41 ng/ml of lactoferrin on collagen I coated dishes to 47.43 to 61.56 ng/ml of scIgA, 24.36 to 144.74 ng/ml of lysozyme and 32.45 to 40.31 ng/ml of lactoferrin on Matrigel™ coated dishes (Table 8) in day 7 cultures. These cultures retain their secretory ability even till day 21. As anticipated, the amount of protein secretion tends to increase from day 7 to day 14 of *in-vitro* culture and then declines by day 21 (Table S2). The possible reason for this could be that by day 14 the cultures tend to attain confluence and so there are more number of acinar cells secreting the proteins; however post confluence the cells tend to reduce the amount of protein secretion either because on passaging these cells they acquire a more fibroblastic morphology or if they are left unpassaged they stop proliferating and hence protein synthesis also reduces. However, it was noted that the level of proteins secreted by cultures growing on Matrigel remains the highest in all the three cases. An interesting observation made here is that the *in-vitro* pattern of secretion of these proteins is the same as that seen *in-vivo* i.e. Lysozyme>scIgA>Lactoferrin [37].We do observe a wide range in the quantity of proteins secreted *in-vitro*. One of the possible reasons for this could be the experimental variables like the age of donor tissue. In the present study, tissue was harvested from exenterated specimens of patients with an age range of 3 years to 65 years. Since the lacrimal protein secretions tend to reduce with increasing age we feel this could be an important factor contributing to such a wide range in quantity of protein secreted *in-vitro*.

Both lacrimal and salivary gland acinar cells share similar developmental, morphologic and functional characteristics and show similar reduction in function when injured [38], [39], [40] but unlike the salivary gland where there is evidence of stem/progenitor cell compartment in the terminal ductioles [17], such evidence is restricted to one preliminary report of stem cells in the mouse lacrimal gland which can be recruited to repair damage to the gland and which can subsequently be cultured in-vitro [24]. Our results give preliminary evidence indicating that the stem-like cells are present in the lacrimal gland but their exact locations is still under investigation. The expression of these stem-like cells in the native tissue could range between 3.1±0.61% (ABCG2 expression) to 3.8%±1.26% (high ALDH1 expression) and between 0.3±0.15% (ABCG2 expression) to 2.7±1.54% (ALDH1 high expression) after 14–18 days of *in-vitro* culture. It is noteworthy that our culture system could maintain stem cells in-vitro even by day 21 though their numbers reduced to 0.2±0.13% (ABCG2) to 1.1±0.5% (ALDH1 high expression). The results of the immunohistochemistry experiments indicate that acinar cells which show strong positivity for vimentin could be the stem cells. However, for the lack of substantiating data, at the present time, no concrete inference can be drawn.

In order to substantiate the evidence of stem-like/progenitor cells in our culture system, clonal or similar stem cell assays have to be performed either with freshly isolated cells or cells obtained after sorting for the stem cell markers. While animal studies and clone forming ability would have added value to this study, the formation of spherules with ‘duct-like’ structures between them are important novel findings, which leads us to believe that we may be able to simulate an *in-vivo* mimicry under our culture conditions.

Even though dry eye syndrome has a global prevalence the treatment still remains palliative and conservative, with artificial tear substitutes and lubricants forming the main stay of clinical management. A similar spectrum of signs and symptoms exist in the case of dry mouth syndrome (xerostomia) caused due to damage to the salivary gland. This damage, similar to lacrimal gland, can be due to aging, hormonal imbalance or radiotherapy induced. The management strategy in such cases used to be palliative but recent reports in literature show the reversal of radiation-induced xerostomia with cell therapy in animal models [34]. The success obtained in these animal models of xerostomia has now shifted the focus towards exploring similar options, especially in conditions with irreparable damage to the lacrimal gland tissue.

In summary, this study provides the first evidence for the successful growth of fresh human lacrimal gland tissues *in-vitro* with an attempt towards duct-like formation and retained secretory function. The study also gives the first preliminary evidence for the presence of stem-like cells in the native human lacrimal gland tissue and these can be maintained under *in-vitro* condition. Further validation of this data would allow the development of a functionally competent 3 D construct for potential clinical application in severe cases of radiation induced dry eye.

## Supporting Information

Figure S1 **Pattern of epithelial cell growth on uncoated, HAM, collagen and Matrigel™ coated dishes.**
(TIF)

Table S1 **Flow cytometric evaluation of cell population growing on uncoated, collagen coated and Matrigel™ coated dishes.**
(DOC)

Table S2 **Tear protein secretion on various matrices post carbachol secretion on day 7, day 14 and day 21.**
(DOC)
