# Supplementary figures and images for: Establishing Human Lacrimal Gland Cultures with Secretory Function
Source: PLoS One. 2012 Jan 13;7(1):e29458. doi: 10.1371/journal.pone.0029458 (PMC3258235; doi:10.1371/journal.pone.0029458)

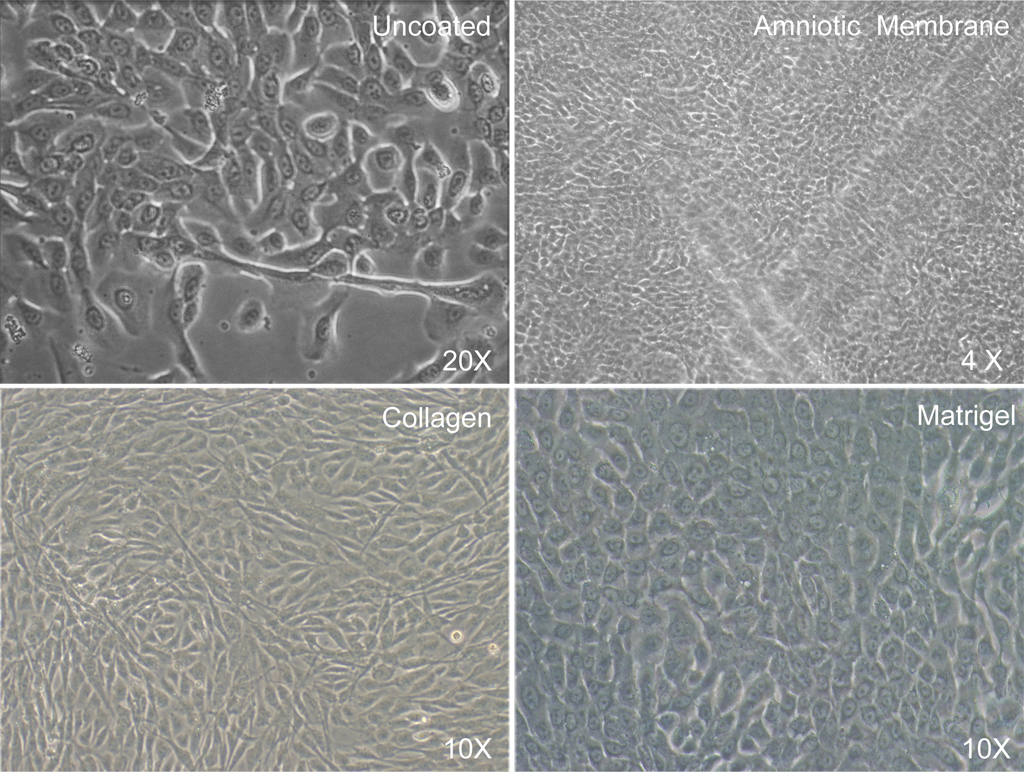

Supplement: Figure S1 — Pattern of epithelial cell growth on uncoated, HAM, collagen and Matrigel™ coated dishes. (TIF) [file pone.0029458.s001.tif]
