# Supplementary material for: Establishing Human Lacrimal Gland Cultures with Secretory Function
Source: PLoS One. 2012 Jan 13;7(1):e29458. doi: 10.1371/journal.pone.0029458 (PMC3258235; doi:10.1371/journal.pone.0029458)
Supplement: Table S1 — Flow cytometric evaluation of cell population growing on uncoated, collagen coated and Matrigel™ coated dishes. (DOC) [file pone.0029458.s002.doc]

| S. No | Marker | Uncoated  (Mean ± SEM) | Collagen 1  (Mean ± SEM) | Matrigel™  (Mean ± SEM) |
| --- | --- | --- | --- | --- |
| 1. | EpCAM | 0.65 ± 0.35 | 1.2 ± 0.3 | 2.2 ±1.70 |
| 2. | CD 90 | 85.1 ± 4.9 | 16.7 ± 0.85 | 13.3 ± 10.20 |

Supplementary Table 1
