# Supplementary material for: Establishing Human Lacrimal Gland Cultures with Secretory Function
Source: PLoS One. 2012 Jan 13;7(1):e29458. doi: 10.1371/journal.pone.0029458 (PMC3258235; doi:10.1371/journal.pone.0029458)
Supplement: Table S2 — Tear protein secretion on various matrices post carbachol secretion on day 7, day 14 and day 21. (DOC) [file pone.0029458.s003.doc]

| Days in culture → | 7 | | | 14 | | | 21 | | |
| --- | --- | --- | --- | --- | --- | --- | --- | --- | --- |
| Tear Proteins ↓ | HAM  (ng/ml) | Collagen  (ng/ml) | Matrigel™  (ng/ml) | HAM  (ng/ml) | Collagen  (ng/ml) | Matrigel™  (ng/ml) | HAM  (ng/ml) | Collagen  (ng/ml) | Matrigel™  (ng/ml) |
| Lysozyme | 5.78 to 33.94 | 0.21 to 18.34 | 24.36 to 144.74 | 133.7 to150.5 | 104.0 to 125.9 | 183.3 to 394.8 | 3.9 to 70.7 | 75.2 to 86.2 | 93.5 to 103.3 |
| ScIgA | 3.86 to 71.40 | 1.41 to 27.58 | 47.43 to 61.56 | 193.6 to 321.2 | 186.5 to 190.1 | 336.4 to 389.3 | 2.71 to 20.5 | 164.5 to 168.2 | 172.8 to 178.6 |
| Lactoferrin | 44.50 to 45.65 | 28.52 to 30.41 | 32.45 to 40.31 | 46.3 to 63.7 | 39.6 to 41.5 | 67.5 to 71.8 | 3.34 to 4.8 | 9.2 to 23.6 | 23.9 to 27.5 |

Supplementary Table 2
